# A Case of New Onset Diabetes and Severe Diabetes Ketoacidosis in a Patient With COVID-19

**DOI:** 10.7759/cureus.16923

**Published:** 2021-08-05

**Authors:** Latika Patel, Sarah Ayad, Mohammad Nabil Rayad, Kirolos Gergis, Chidinma Ejikeme, Afrah Talpur, Basel Abdelazeem, Ari Eckman

**Affiliations:** 1 Internal Medicine, Rutgers-New Jersey Medical School/Trinitas Regional Medical Center, Elizabeth, USA; 2 Internal Medicine, Rutgers-New Jersey Medical School/ Trinitas Regional Medical Center, Elizabeth, USA; 3 Internal Medicine, St. Michaels Medical Center, Newark, USA; 4 Internal Medicine, McLaren Health Care, Flint, USA; 5 Internal Medicine, Liaquat University of Medical and Health Sciences, Jamshoro, PAK; 6 Internal Medicine, McLaren Flint/Michigan State University, Flint, USA; 7 Internal Medicine and Endocrinology, Rutgers-New Jersey Medical School/ Trinitas Regional Medical Center, Elizabeth, USA

**Keywords:** covid-19, sars-cov-2, diabetic ketoacidosis (dka), hyperglycemia, hemoglobin a1c, diabetes typee i, diabetes type ii.

## Abstract

Diabetic ketoacidosis (DKA) is a significant complication of poorly controlled diabetes. In diabetics, it typically occurs due to insulin deficiency resulting in lipolysis and subsequent ketone body formation and acidosis. The emergence of the COVID-19 infection has been associated with several complications, with the most prominent being pulmonary and cardiovascular-related. However, in some cases, patients with COVID-19 infection present with diabetic ketoacidosis. The pathophysiology of DKA in COVID-19 infection is different and currently not completely understood. The manifestation of DKA in COVID-19 patients is associated with increased severity of mortality and length of stay in these patients. Here, we describe a patient with no past medical history who presented with COVID-19 symptoms and was found to be in DKA. This case report highlights the possible underlying pathophysiology associated with this complication.

## Introduction

Diabetic ketoacidosis (DKA) is a serious acute complication of uncontrolled diabetes, characterized by hyperglycemia and ketoacidosis. DKA is more commonly seen in patients with type 1 diabetes however, it is also being seen in higher frequencies in patients with type 2 diabetes [1].

Over the past few decades, there has been a large increase in hospitalization rates from DKA which may be related to the increased prevalence of type 2 diabetes. DKA occurs due to the presence of insulin deficiency which can lead to unregulated glucose levels and lipolysis levels. This in turn leads to the formation of ketone bodies and acidosis [2]. DKA is known to be an inflammatory state that is often caused by an underlying infection or severe illness [2].

Severe acute respiratory syndrome coronavirus 2 (SARS-CoV-2) causes COVID-19 pneumonia as well as extrapulmonary complication. There are a few cases of precipitation of new-onset diabetes and diabetes ketoacidosis associated with COVID-19 [3,4]. We present a case of a 44-year-old patient with COVID-19 and concomitant new onset of diabetes and severe diabetes ketoacidosis.

## Case presentation

A 44-year-old Hispanic female with no prior history of diabetes and recent diagnosis of COVID-19 was brought into the emergency room by ambulance due to worsening fatigue and myalgias for the last three days. She reported that she tested positive for COVID-19 on reverse transcription-polymerase chain reaction (RT-PCR) assay three days prior to presentation and her condition had been progressively getting worse. She also reported subjective fever, chills, exertional dyspnea, and labored breathing. She did not seek medical care for COVID-19 up to that point. The patient also reported dry cough and extreme thirst over the past two to three days. She had been drinking cups and cups of water, but still felt very dehydrated. She denied chest pain and palpitation. She also denied abdominal pain, nausea, and vomiting.

On arrival to the emergency department (ED), the patient was afebrile (98.5 F), tachycardia (heart rate 136/min), tachypneic (respiration rate 40/min), hypertensive (blood pressure 142/88 mm-Hg), and oxygen saturation was 94% on 3 liters nasal cannula. Physical examination revealed an age-appropriate female on oxygen supplementation via nasal cannula who was in moderate apparent respiratory distress. Lung examination showed bilateral diffuse rhonchi with decreased breath sounds at bases. The rest of the physical exam was unremarkable.

**Figure 1:**
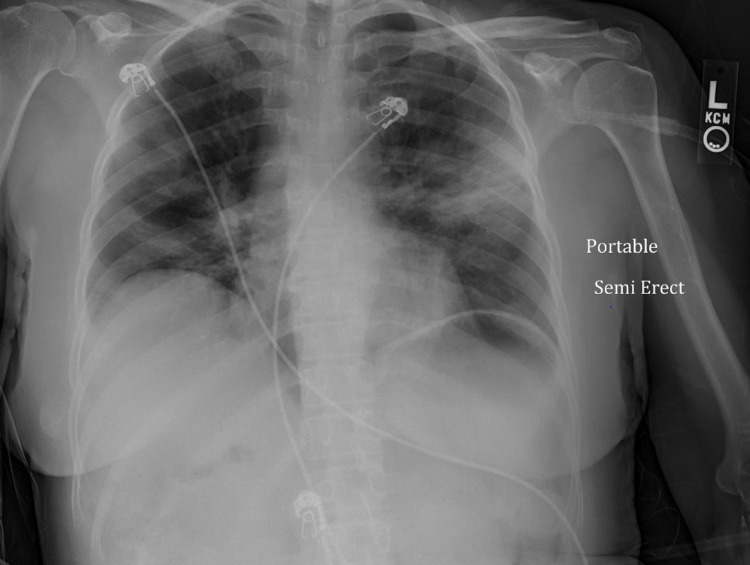
Chest radiograph revealed bilateral opacities

**Figure 2:**
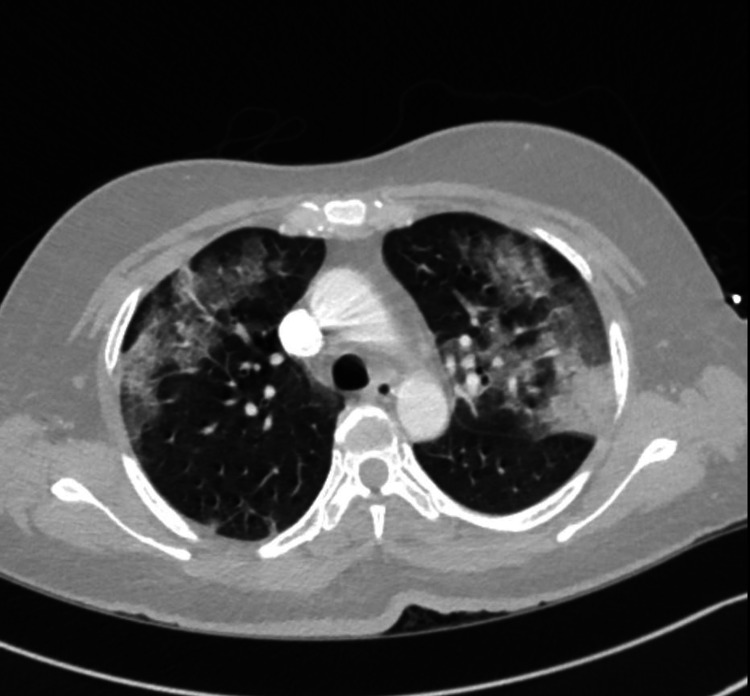
CT angiography showed diffuse bilateral ground glass opacities consistent with COVID 19 pneumonia

On investigation, chest radiograph revealed bilateral opacities as seen in (Figure 1). CT angiography showed diffuse bilateral ground-glass opacities consistent with COVID-19 pneumonia (Figure 2). Electrocardiogram showed sinus tachycardia with possible left atrial enlargement.

Laboratory investigations revealed random plasma glucose of 426 mg/dL, moderate acetone, anion gap 24 and potassium of 3.4 mmol/l(Table 1). Venous blood gas analysis on a 3 L nasal cannula indicated severe metabolic acidosis (pH 6.93 and HCO 3-2.8 mmol/L) (Table 2). Urine ketones were present. 

**Table 1:** Relevant laboratory values

Comprehensive metabolic panel	Reference range	Value on presentation
Creatinine	0.4-1 mg/dl	1.08
Urea Nitrogen	8-20 mg/dl	12
Glucose Level	74-118 mg/dl	426
Calcium Level	8.9-10.3 mg/dl	7.6
Albumin Level	3.5-4.8 g/dl	2.9
Sodium Level	136-144 mmol/l	139
Potassium Level	3.6-5.1 mmol/l	3.4
Chloride Level	101-111 mmol/l	110
Total Co2	22-32 mmol/l	4
Anion GAP (AGAP)	3-9 mmol/l	24

**Table 2:** Table 2 venous blood gas analysis

Arterial blood gas	Reference range	Value on presentation
Mode		BIPAP
Fio2		35%
IPAP		10
EPAP		5
PH	7.35-5.45	6.93
PCO2	35-45 mmHg	13
PO2	>80 mmHg	109
HOC3	22-26 mmol/L	2.8
O2 saturation	>90 %	98.2

These findings confirmed the diagnosis of severe diabetes ketoacidosis. The management was initiated with initial intravenous fluid, electrolytes replacement, and intravenous (IV) bicarbonate push followed by intravenous insulin infusion as per DKA protocol. Serum electrolytes were closely monitored. Lovenox 60 mg subcutaneous twice daily for DVT prophylaxis. Decadron was not started for COVID-19 as the risk of exacerbating DKA outweighed the benefits of providing an anti-inflammatory effect against COVID-19. 

On day three of hospitalization, a laboratory study was significant for severe hypokalemia (potassium of 2.7) and potassium was aggressively replenished. The patient’s hospital course got complicated due to acute respiratory failure requiring mechanical ventilation. Remdesivir therapy was initiated for COVID-19 viral pneumonia. 

The patient’s DKA resolved on hospital day four and successfully transitioned to subcutaneous insulin therapy. Ten days later, she was successfully liberated from mechanical ventilation to venti mask 40% Fio2. She was weaned off oxygen and discharge home with Aspart 3 units three times with meal and 12 units Detemir at bedtime and 6 Units Detemir in the morning. She was given follow-up with the medical clinic after one month.

## Discussion

COVID-19 is associated with severe complications that are frequently seen in those that have multiple comorbidities including cardiovascular disease, hypertension, obesity, and diabetes. Acute hyperglycemic crises can be broken down into two categories: diabetic ketoacidosis (DKA) and hyperosmolar hyperglycaemic state (HHS). The onset of these hyperglycemic crises with concomitant COVID-19 infection can present a challenge in terms of management [5]. We presented a case of new-onset diabetes presenting as DKA precipitated by COVID-19 infection. 

The relationship between DKA and COVID-19 has been explored sparingly thus far in the literature. In a study by Li et al., that included 658 hospitalized patients with confirmed COVID-19 infection, 42 of them presented with ketosis on admission. These patients were more likely to have a longer hospital stay and higher mortality rate [6]. Within the group of 42 ketosis-positive patients, 27 did not have diabetes and 15 of them did. Only three of the patients with diabetes and ketosis had evidence of ketoacidosis. Thus, COVID-19 infection is capable of inducing DKA. Interestingly, the relationship between DKA and COVID is bidirectional. Patients who have DKA are at higher risk of developing severe COVID-19 due to the co-morbid diabetic condition which places these patients at a higher risk [5]. On the other hand, patients that have COVID-19 are oftentimes on glucocorticoid therapy can induce hyperglycemia. And, COVID-19 can also worsen glycemic control in both new-onset diabetic patients and previously diabetic patients [7].

The relationship between DKA and COVID-19 is not completely understood but there have been proposed explanations. Firstly, it can be explained by the presence of pro-inflammatory cytokines which are seen in both conditions. Specifically, interleukin-6 (IL-6) is seen to be elevated in DKA and COVID-19. In COVID-19, elevated levels have been linked to an insufficient immune response against the virus. It has become an important prognostic factor in COVID-19 patients and is a target of therapy [2]. Another mechanism that may explain the interplay between DKA and COVID-19 is related to the renin-angiotensin-aldosterone system (RAAS). Angiotensin-converting enzyme 2 (ACE2) is found in the lungs and pancreas and is responsible for the conversion of angiotensin II to angiotensin. Since it is expressed in the lungs, it may serve as a target where the virus can enter from. Once the virus enters and goes to the pancreatic B cells, it can directly cause injury to the cells which are responsible for producing insulin. Furthermore, in COVID-19 infection, ACE2 levels are downregulated once the virus becomes endocytosed. This results in high levels of angiotensin II which can inhibit the secretion of insulin [8]. Overactivation of the RAAS system can trigger a state of insulin resistance due to an inability to uptake glucose and oxidative stress due to reactive oxygen species (ROS) production [9].

The management of these patients is by treating diabetic ketoacidosis based on protocols. It includes initiating insulin therapy, managing fluid status, and potassium replacement and correction as needed [2]. Fluid management is usually done with isotonic saline (0.9% NaCl). Complicated patients with COVID-19 generally require intravenous therapy with insulin. Management guidelines for COVID-19 patients with DKA have not yet been established; however, it has been reported that COVID-19 patients with DKA require higher than usual insulin levels [10]. Another important consideration is for the concomitant COVID-19 infection. One of the approved treatments is a course of corticosteroid therapy. This is not possible in these patients due to their already elevated glucose levels [10]. Thus, the management of both COVID-19 and DKA together may pose a challenge. 

## Conclusions

Overall, this case highlights the importance of being vigilant and carefully treating patients who present with both COVID-19 and DKA since there are higher mortality rates in those patients. Furthermore, the mechanism of COVID-19 induced ketosis is still not well understood and requires further research.
